# Annotations

**Published:** 1902-05-31

**Authors:** 


### Annotations.

### A Lesson in Humility.

The terrible events which have taken place during the past fortnight in the West Indies have served to remind us of the ceaseless and unrelenting operation of cosmic laws and of the littleness of man, and as members of a profession largely engaged in a struggle against these laws and in attempts to save individuals from penalties imposed upon them by a more or less outraged Nature we may well learn a lesson in humility. How futile all our efforts seem! We pride ourselves on the lives we save by removing tumours and deformities. Yet if we are to accept the general principles of evolution we are but propping up those who ought to die, and encouraging the upspringing of other generations which at the cost of still more suffering will have to be eliminated. With infinite pains and no small self-congratulation we track out the origin of enteric fever, and to a large extent protect our population from its ravages, and then to our sorrow we discover that the carefully protected people of the present generation are more susceptible than ever to the infection. We introduce western forms of civilisation into India, we abolish suttee, give peace, put an end to infanticide, and feel that we have done great things, only to find that population has increased, that overcrowding and close living have brought their own dangers, and that people so bred and so housed are ravaged by plague—a penalty we had forgotten all about—and that in the presence of this calamity we have to stand helpless. By aid of many hospitals and a widespread sanitary organisation we lower by a small percentage the death rate in this country, and we speak as if each scrofulous infant saved from an early grave, and each feeble old man enabled to live a few more weary years upon the charity of his fellows were an addition to England's potential greatness. Meanwhile, Nature steps in and quietly undoes all our handiwork by lowering the birth rate. A nation is great, not by the number of its population, but by its fertility, its power of filling up vacancies, and making good its losses whenever they occur, and herein has always been England's greatness. Yet that is where we now are failing. We, the doctors, are elated because here and there we save a life. But of what good if lives cease to be produced? What some call Nature, what others speak of as cosmic law is relentless and omnipotent; we are but flies upon the wheel.

### Lunacy Law Reform.

We hear much from time to time concerning the increase of lunacy, and many attempts have been made to explain away the statistics by pointing out that the increase is apparent only, being due to the larger proportion of the insane who now flock to the asylums rather than to any actual increase in the total number affected. This may be so, but no one who looks into the matter can fail to see that a very large number of persons of unsound mind still remain outside the provisions of the lunacy laws. While the poor are becoming increasingly willing to accept the hospitality of the asylums, the spread of education and the constant references to the subject of heredity in the public press have led an increasingly large number of people to dread the stigma of insanity. Thus it happens that they endeavour at all hazards to keep their insane relatives outside the pale of legal insanity. Again there can be no doubt that the stringency of the conditions under which lunacy certificates can be obtained, by rendering the placing of a relative in an asylum a somewhat complicated and tedious process, tempts people, so long as there seems any hope of the attack blowing over, to shirk responsibility by handing the patient over to anyone who will take care of him for a time. Added to all this there is the growing reluctance of medical men to sign certificates, unless the case be one of obvious and pronounced insanity, on account of the liability to trouble and loss should the patient upon recovering turn out to be of a litigious disposition. These various causes are quite sufficient to account for the by no means inconsiderable number of lunatics who at the present time are either at large or are being detained in a manner which, however convenient it may be, is absolutely contrary to law. Under the pretence of being "slight nervous cases" or "neurasthenics" or "drug cases," people of unsound mind, often in the early stages at which proper treatment might be followed by the best results, are placed in unlicensed and uninspected "homes" of various sorts in which they are kept under far more restraint than they would be in properly licensed asylums. All this will have to be taken into consideration whenever any serious attempt is made to secure a revision of the lunacy laws. Something must be done to deal with "borderland" cases and to give them the same protection that is by law provided for the confessedly insane.

### Experiments on Animals.

The annual report of the inspectors on the experiments on living animals performed during the year 1901 under license from the Home Office has just been issued as a Parliamentary paper. From it we gather that in England and Scotland there were 257 licensees, of whom 56 performed no experiments during the year, and that "licenses and certificates have been granted and allowed only upon the recommendation of persons of high scientific standing; that the licensees are persons who, by their training and education, are fitted to undertake experimental work, and to profit by it; and that all experimental work has been conducted in suitable places." In reference to the groundless but frequently repeated assertions made by ignorant but self-confident people in regard to the similarity between the experiments on animals as conducted in England, and certain operations from time to time reported from abroad, it is satisfactory to learn from this return that "in no case has a cutting operation more severe than a superficial venesection been allowed to be performed without anæsthetics." It is important to note in this connection that a very large number of the inoculation experiments which were performed without anæsthetics were not done in the course of physiological investigations, but were undertaken for the diagnosis or treatment of disease, or were necessitated by the requirements of the public health authorities, 2,636 experiments having been thus performed for the testing of antitoxin, and 2,985 in various other official investigations, more than half of which had to do with the testing of milk.

